# Skin/Muscle Incision and Retraction Induces Evoked and Spontaneous Pain in Mice

**DOI:** 10.1155/2019/6528528

**Published:** 2019-07-31

**Authors:** Juan Yang, Fei Yuan, Gang Ye, Yong-Jie Wang, Cheng Wu, Jinghua Wang, Xiang-Yao Li, Zhiying Feng

**Affiliations:** ^1^Department Pain Medicine, The First Affiliated Hospital, School of Medicine, Zhejiang University, Hangzhou, Zhejiang 310003, China; ^2^Department of Anesthesiology & Pain Medicine, Zhejiang Provincial Hospital of Traditional Chinese Medicine, Hangzhou, Zhejiang 310006, China; ^3^Department of Anesthesiology & Pain Medicine, Shaoxing Second Hospital, Shaoxing, Zhejiang 312000, China; ^4^Department of Anesthesiology & Pain Medicine, Shaoxing People's Hospital, Shaoxing, Zhejiang 312000, China; ^5^Institute of Neuroscience, Key Laboratory of Medical Neurobiology of the Ministry of Health of China, School of Medicine, Zhejiang University, Hangzhou, Zhejiang 310058, China

## Abstract

**Background:**

Surgery is a frequent cause of persistent pain. Unrelieved chronic postsurgical pain causes unnecessary patient suffering and discomfort and usually leads to psychological complications. The rat model of skin/muscle incision and retraction (SMIR) with decreased paw withdrawal thresholds developed by Flatters was usually used to investigate the underlying mechanism of chronic postsurgical pain.

**Objectives:**

The aim of our study was to develop a new mice model of SMIR for further investigation with transgenic mice and so on and to evaluate the analgesic effects of clonidine and gabapentin on pain behavior with this new mice model.

**Methods:**

Male C57BL/6 mice were anesthetized, and a 1.0–1.3 cm incision was made in the skin of the medial thigh approximately 3 mm medial to the saphenous vein to reveal the muscle of the thigh. The paw withdrawal threshold (PWT) to mechanical stimuli and the paw withdrawal latency to heat stimuli were measured before and after SMIR. Furthermore, the PWT to mechanical stimuli and conditioned place preference (CPP) was measured before and after the systemic injection of clonidine and gabapentin.

**Results:**

SMIR-evoked mechanical hypersensitivity in mice began on day 1 after the procedure, prominent between days 1 and 10 after the procedure, persisted at least until day 14, and disappeared on day 18 after the procedure. However, the mice model of SMIR did not evoke significant heat hypersensitivity. Systemic injection of clonidine and gabapentin raised the PWT in the SMIR mice dose-dependently. Compared with the mice that underwent the sham operation, mice of SMIR spent a longer time in the clonidine-paired chamber than those of NS, while the gabapentin-paired chamber has no difference with that of NS in the CPP paradigm.

**Conclusion:**

These data suggested that the mice model of SMIR demonstrated a persistent pain syndrome, including evoked pain and spontaneous pain. Clonidine and gabapentin could relieve mechanical hypersensitivity dose-dependently simultaneously. However, clonidine but not gabapentin could alleviate the spontaneous pain of SMIR in the mice model.

## 1. Introduction

Since the first definition of chronic postsurgical pain (CPSP) by Macrae [1] in 1999, the phenomenon has been recognized increasingly. About 10–50% of patients suffer such persistent postsurgical pain, despite advances in surgical techniques and perioperative analgesic strategies [1–5]. It has been well reported that spontaneous pain, allodynia, and hyperalgesia are major problems for patients with persistent pain [6–9]. Unrelieved CPSP causes unnecessary patient suffering and discomfort and leads to psychological and pathophysiological complications. The CPSP has become a separate category in the latest IASP classification of pain, to be included in the ICD-11.

All of these procedures involve essential and prolonged tissue retraction, which could account for the persistent nature and high incidence of postsurgical pain. A new model of persistent postsurgical pain evoked by skin/muscle incision and retraction (SMIR) in rats invented by Flatters [10] was used widely for investigating the underlying mechanism of CPSP [11–13]. Therefore, developing a new SMIR model in mice is necessary for further investigation with transgenic mice and so on. Clonidine, a specific alpha-2 adrenergic receptor agonist, has a well-established analgesic profile. It has been found to have a wider application as an adjunct to anesthetics and analgesics in perioperative settings [14–17]. It can relieve mechanical allodynia in oxaliplatin-induced neuropathic mice model [18, 19]. Gabapentin, an antiepileptic drug and structural analogue of the neurotransmitter gamma-aminobutyric acid, was developed as an anticonvulsant and subsequently used for various chronic pain conditions [20–22]. However, the effects of the clonidine and gabapentin on the spontaneous pain, allodynia, and hyperalgesia in the model of SMIR have not been investigated until now.

The aim of this study was to characterize a mice model of CPSP through skin and muscle incision and retraction with pain behavior including conditioned place preference (CPP) paradigm to assess spontaneous pain. We also evaluated the different analgesic effects of clonidine and gabapentin on the new model.

## 2. Methods

### 2.1. Animals and Surgery

Adult C57/BL6 mice (8 weeks, 25–30 g) were purchased from the Experimental Animal Center of Zhejiang University. All animal procedures in this study were performed according to the guidelines of the International Association for the Study of Pain and were approved by the Animal Care and Use Committee of Zhejiang University.

**Figure 1:**
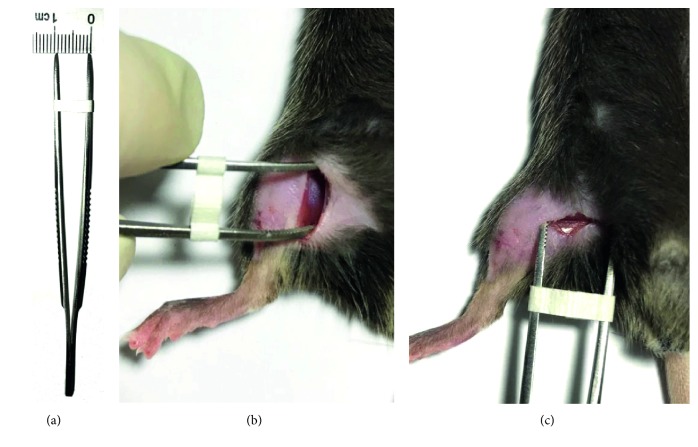
Photograph of injury site during the 1-hour retraction period of skin/muscle incision and retraction (SMIR) surgery. (a) Custom-made retractor with a spreading distance of 1 cm. (b) Revealing the fascia of the underlying adductor muscles. (c) The muscles were parted by the custom-made retractor for 1 hour.

To produce SMIR, animals were anesthetized with sodium pentobarbital (40–50 mg/kg, intraperitoneally (i.p.)), laid on their back, and the medial thigh on the right side was shaved. The shaved skin was then repeatedly swabbed with sterile alcohol wipes to sterilize the area and to allow visualization of the saphenous vein. A 1.0–1.3 cm incision was made in the skin of the medial thigh, approximately 3 mm medial to the saphenous vein, to reveal the muscle of the thigh. An incision (about 1 cm long) was then made in the superficial (gracilis) muscle layer of the thigh, approximately 3 mm medial to the saphenous nerve. The superficial muscle was then parted further, by spreading blunt scissors into the muscle incision site, to allow the insertion of a custom-made dissecting retractor. The skin and superficial muscle of the thigh were then retracted by 1 cm, revealing the fascia of the underlying adductor muscles; this retraction was maintained for 1 hour. Sham-operated mice underwent the same procedure with the exception of the skin/muscle retraction (Figures 1(a)–1(c)). Following recovery from anesthesia, all animals could ambulate normally and rise up on their hindpaws to reach food and water.

### 2.2. Mechanical Allodynia Test

On the experimental day, the von Frey behavioral test was performed according to the up-down algorithm described by Chaplan et al. [23]. To determine evoked reflex responses to mechanical stimuli, animals were placed on a raised mesh grid and covered with a clear plastic box. Calibrated von Frey filaments were applied to the middle of the plantar surface of the right paw until the filament bent. Brisk withdrawal or paw flinching was considered as a positive response. Lifting of the paw due to normal locomotor behavior was ignored. In the absence of a response, the filament of the next greater force was applied. If a response was obtained, the filament of the next lower force was applied. The tactile stimulus producing a 50% likelihood of withdrawal response was calculated and treated as the paw withdrawal threshold (PWT).

### 2.3. Heat Allodynia Test

Animals were placed in individual Perspex boxes on a glass floor. Nociceptive responses to a noxious heat stimulus were examined by measuring the hindpaw withdrawal latency (PWL) from a focused beam of radiant heat projected to the plantar surface (Ugo Basile Plantar Test apparatus, Gemonio, Italy). The withdrawal latency to this stimulus was measured in seconds, and the apparatus had a built-in cutoff latency of 20 seconds. The operated hindpaw of each mouse was tested three times and then the average of these three readings was taken. The heat sensitivity on separate days and then on days 1, 3, 7, 10, 14, and 18 after surgery was determined.

### 2.4. Conditioned Place Preference Protocol

CPP was adapted from the behavioral paradigm established by King et al. in adult rats [24, 25]. CPP testing was conducted using three Plexiglas chambers separated by manual doors. The two end chambers were connected via one center chamber. The walls of one chamber were white with horizontal black stripes and the walls of the other chamber were white with vertical black stripes. We used the chambers with striped walls to ensure that mice would not strongly prefer one chamber to the other.

Habituation was performed across 2 days for 30 minutes each day; mice were permitted to move freely to all chambers. On day 3, a preconditioning preference test was conducted to determine whether a chamber bias existed. Mice were placed into the middle chamber and permitted to move freely in all chambers for 15 minutes, and the time spent in each end chamber was recorded. The mice that spent more than 80% or less than 20% of the total time in a single chamber were eliminated from further study. On day 4, mice received vehicle (e.g., saline) chamber pairing and drug pairing 4 hours later. During conditioning, mice were allowed to stay only in the paired chamber, without access to other chambers for 15 minutes immediately following the vehicle or drug injection, including clonidine and gabapentin. Clonidine and gabapentin were purchased from Sigma-Aldrich (St Louis, MO, USA). Chemicals were dissolved in sterile saline. Different volumes of stock solution were administered to the animals i.p. with a 1 ml syringe. Twenty hours after drug pairing, mice were placed in the middle chamber of the CPP box with all doors open, so that the animals could have free access to all chambers. The time spent in the drug-paired chamber and saline-paired chamber was calculated. The preference index was calculated as the time spent in the drug-paired chamber subtracted from the time spent in the saline-paired chamber.

### 2.5. Statistical Analyses

GraphPad Prism 7.0 was used to plot and fit the data. Statistical comparisons were made using Student's *t*-test, paired *t*-test, and two-way repeated-measures ANOVA (Student–Newman–Keuls test was used for post hoc comparison). All data are presented as the mean ± SEM. In all cases, *P* < 0.05 was considered statistically significant.

## 3. Results

### 3.1. Mouse Model of Persistent Postsurgical Pain Evoked by SMIR

**Figure 2:**
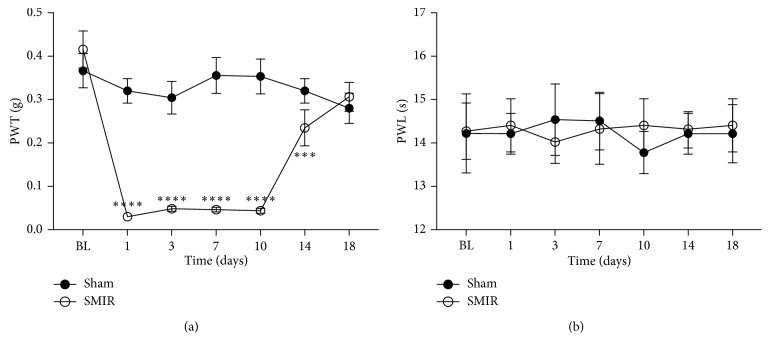
Paw withdrawal threshold (PWT) for mechanical stimulation and paw withdrawal latencies (PWLs) to noxious heat stimulation evoked by skin/muscle incision and retraction (SMIR) surgery. (a) The sham treatment group did not demonstrate significant mechanical hypersensitivity as compared to baseline, to von Frey stimulation in the ipsilateral paw. SMIR surgery evoked a persistent significant mechanical hypersensitivity. SMIR-evoked mechanical hypersensitivity in the ipsilateral paw (two-way RM ANOVA, sham versus SMIR: F1; 16 = 50.6, *P* < 0.0001; time: F6; 96 = 15.42, *P* < 0.0001, interaction: F6; 96 = 15.48, *P* < 0.0001; *n* = 9 for the sham group, *n* = 9 for the SMIR group; ^*∗∗∗*^*P* < 0.001, ^*∗∗∗∗*^*P* < 0.0001 under Sidak's multiple comparisons test). (b) Paw withdrawal latencies in the sham-operated group were unaltered throughout the time course. SMIR surgery also did not evoke significant heat hypersensitivity. Withdrawal latencies of the ipsilateral paws in the SMIR-operated mice were not significantly altered up to postoperative day 18 (two-way RM ANOVA, sham versus SMIR: F1; 17 = 0.013, *P*=0.91; time: F6; 102 = 0.07, *P*=0.999, interaction: F6; 102 = 0.23, *P*=0.97; *n* = 9 for the sham group, *n* = 10 for the SMIR group, under Sidak's multiple comparisons test). ANOVA, analysis of variance; RM, repeated measures; BL, presurgery baseline.

SMIR was performed on the right hindpaw of mice (Figures 1(a)–1(c)), and PWTs and PWLs of the ipsilateral hindpaw plantar surface were tested. As compared to the sham-operated mouse, SMIR treatments decreased PWTs on postsurgical day 1 and lasted to day 10, while it recovered at day 18 after SMIR surgery (Figure 2(a)). Sham treatments did not change the PWTs. These data suggested that the SMIR treatments induced mechanical allodynia in mice.

The effect of SMIR surgery on hindpaw responses to thermal stimuli was also investigated.  Figure 2(b) illustrates the PWL to a noxious heat stimulus prior to and up to 18 days after SMIR or sham surgery. Withdrawal latencies of ipsilateral paws in SMIR-operated mice were not significantly altered up to postsurgical day 18. The same findings were made in the sham-operated group.

### 3.2. Clonidine Relieves Evoked Pain and Spontaneous Pain after SMIR

**Figure 3:**
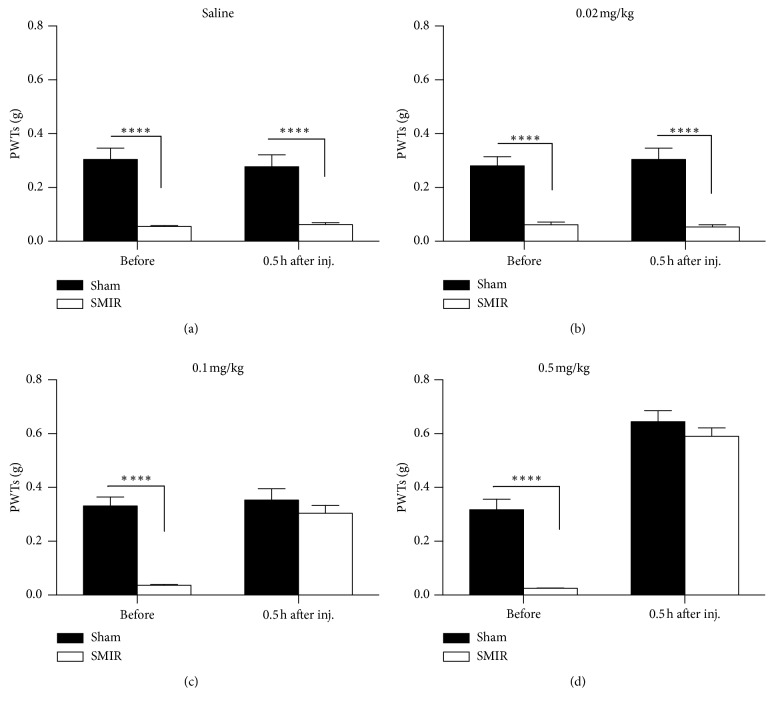
Systemic administration of clonidine raised the paw withdrawal threshold (PWT) in skin/muscle incision and retraction (SMIR) mice. (a) Saline had no effect on the PWTs in the sham and SMIR groups injected at day 4 after SMIR (two-way RM ANOVA, sham versus SMIR: F1; 17 = 35.83, *P* < 0.0001; treatments: F1; 17 = 0.55, *P*=0.47, interaction: F1; 17 = 1.74, *P*=0.20; *n* = 7 for the sham group, *n* = 8 for the SMIR group, ^*∗∗∗∗*^*P* < 0.0001 under Sidak's multiple comparisons test). (b) SMIR decreased the PWTs, which was not changed by the application of clonidine at 0.02 mg/kg (two-way RM ANOVA, sham versus SMIR: F1; 17 = 87.23, *P* < 0.0001; treatments: F1; 17 = 0.08, *P*=0.78, interaction: F1; 17 = 0.33, *P*=0.57; *n* = 9 for the sham group, *n* = 10 for the SMIR group, ^*∗∗∗∗*^*P* < 0.0001 under Sidak's multiple comparisons test). (c) Clonidine at 0.1 mg/kg increased the PWTs of the SMIR group (two-way RM ANOVA, sham versus SMIR: F1; 17 = 24.07, *P*=0.0001; treatments: F1; 17 = 37.01, *P* < 0.0001, interaction: F1; 17 = 26.54, *P* < 0.0001; *n* = 9 for the sham group, *n* = 10 for the SMIR group, ^*∗∗∗∗*^*P* < 0.0001 under Sidak's multiple comparisons test). (d) Clonidine at 0.5 mg/kg increased the PWTs of both the SMIR group and the sham group (two-way RM ANOVA, sham versus SMIR: F1; 17 = 24.04, *P*=0.0001; treatments: F1; 17 = 273.3, *P* < 0.0001, interaction: F1; 17 = 19.53, *P* < 0.0004; *n* = 9 for the sham group, *n* = 10 for the SMIR group, ^*∗∗∗∗*^*P* < 0.0001 under Sidak's multiple comparisons test). ANOVA, analysis of variance; RM, repeated measures.

Clonidine is an agonist of alpha-2 adrenergic receptor; it has had marked analgesic effects on both evoked pain and spontaneous pain in basic research studies; we therefore evaluated the analgesic effects of clonidine on SMIR-induced persistent pain. As shown in Figure 3(a), the injection of saline had no effects on PWTs, while application of clonidine increased the PWTs of mice with SMIR in a dose-dependent manner (Figures 3(b)–3(d), i.p.). More specifically, clonidine increased PWTs at doses of 0.1 mg/kg (Figure 3(c)) and 0.5 mg/kg (Figure 3(d)), but it had no effect at 0.02 mg/kg (Figure 3(b)). Interestingly, clonidine also elevated the PWTs of mice with sham treatments at 0.5 mg/kg, but not at other doses.

**Figure 4:**
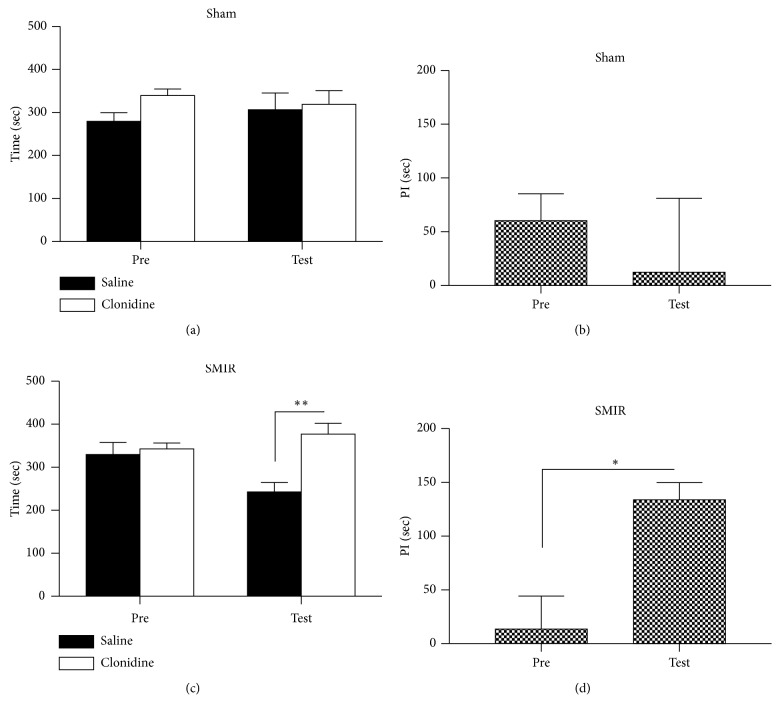
Systemic administration of clonidine induced the place preference to the drug-paired chamber. (a) Mice that received sham treatments spent equal amounts of time in the chambers during the preconditioning and testing periods (two-way RM ANOVA, pre versus test: F1; 23 = 0.06, *P*=0.76; drug versus saline: F1; 23 = 7.54, *P*=0.44, interaction: F1; 23 = 3.29, *P*=0.43; *n* = 6). (b) No difference was detected in the preference times of the sham group (*t*-test, *P* > 0.05). (c) Preference was detected after the application of clonidine (0.5 mg/kg) to the mice of SMIR (two-way RM ANOVA, pre versus test: F1; 23 = 3.41, *P*=0.04; drug versus saline: F1; 23 = 26.81, *P*=0.001, interaction: F1; 23 = 17.99, *P*=0.04; *n* = 6). (d) A significant difference was detected in the preference times of the SMIR group (*t*-test, *P* < 0.05). ANOVA, analysis of variance; RM, repeated measures; PI, preference index.

We further evaluated the effects of clonidine on place preference by using the CPP behavioral paradigm. As shown in Figure 4, mice that received sham treatments did not show place preference to the clonidine-paired chamber during the habitation or testing period (Figures 4(a) and 4(b)), and no difference was detected on the preference index (Figure 4(b)). Mice that underwent the SMIR operation spent a similar amount of time in the two chambers during the habitation period; interestingly, they spent a longer time in the drug-paired chamber during the testing period (Figures 4(c) and 4(d)). These data suggested that SMIR could cause spontaneous pain to occur, which could be relieved by 0.5 mg/kg clonidine.

### 3.3. Gabapentin Relieved the Evoked Pain but Not Spontaneous Pain after SMIR

**Figure 5:**
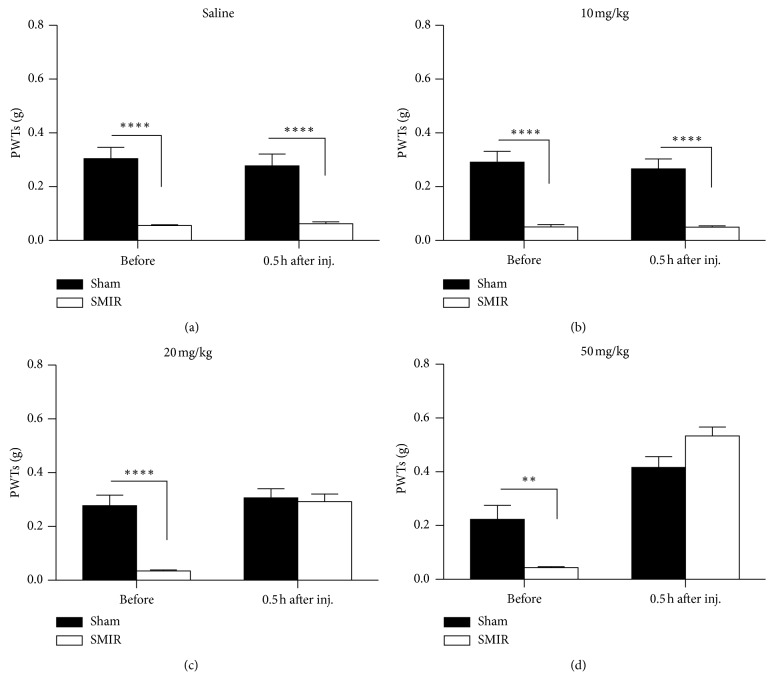
The application of gabapentin systemically raised the paw withdrawal threshold (PWT) in skin/muscle incision and retraction (SMIR) mice. (a) Saline had no effect on the PWTs in the sham and SMIR groups injected at day 4 after SMIR (two-way RM ANOVA, sham versus SMIR: F1; 17 = 35.83, *P* < 0.0001; treatments: F1; 17 = 0.55, *P*=0.47, interaction: F1; 17 = 1.74, *P*=0.20, *n* = 7 for the sham group; *n* = 8 for the SMIR group, ^*∗∗∗∗*^*P* < 0.0001 under Sidak's multiple comparisons test). (b) SMIR decreased the PWTs, which were not changed by the application of gabapentin at 10 mg/kg (two-way RM ANOVA, sham versus SMIR: F1; 17 = 71.68, *P* < 0.0001; treatments: F1; 17 = 0.25, *P*=0.63, interaction: F1; 17 = 0.19, *P*=0.67; *n* = 9 for the sham group, *n* = 10 for the SMIR group, ^*∗∗∗∗*^*P* < 0.0001 under Sidak's multiple comparisons test). (c) Gabapentin at 20 mg/kg increased the PWTs of the SMIR group (two-way RM ANOVA, sham versus SMIR: F1; 17 = 13.41, *P*=0.0019; treatments: F1; 17 = 57.53, *P* < 0.0001, interaction: F1; 17 = 36.69, *P* < 0.0001; *n* = 9 for the sham group, *n* = 10 for the SMIR group, ^*∗∗∗∗*^*P* < 0.0001 under Sidak's multiple comparisons test). (d) Gabapentin at 50 mg/kg increased the PWTs of both the SMIR group and sham group (two-way RM ANOVA, sham versus SMIR: F1; 9 = 0.73, *P*=0.41; treatments: F1; 9 = 102.7, *P* < 0.0001, interaction: F1; 9 = 19.41, *P*=0.0017; *n* = 5 for the sham group, *n* = 6 for the SMIR group, ^*∗∗*^*P* < 0.01 under Sidak's multiple comparisons test). ANOVA, analysis of variance; RM, repeated measures.

**Figure 6:**
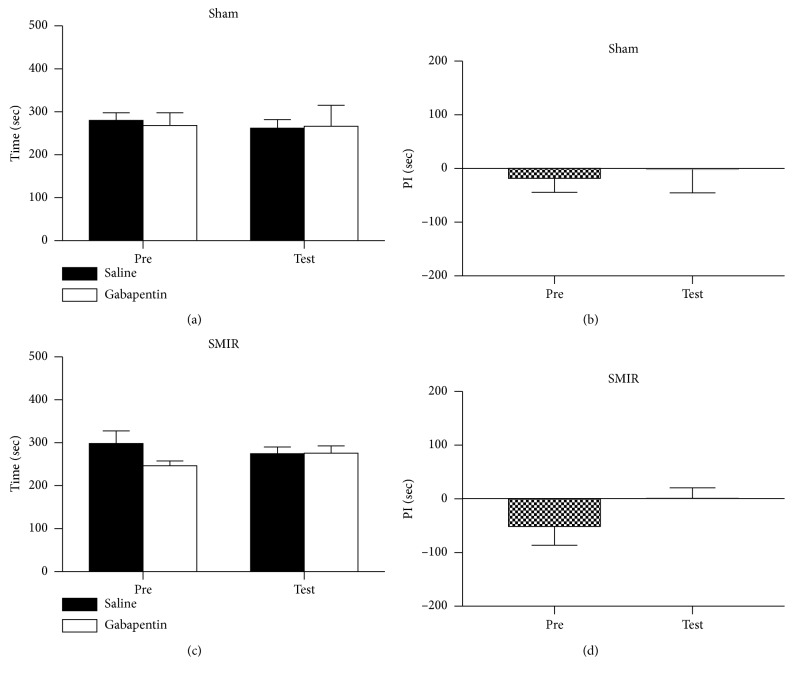
Systemic administration of gabapentin did not induce the place preference to the drug-paired chamber. (a) Mice that received sham treatments spent equal amounts of time in the chambers during the preconditioning and testing periods (two-way RM ANOVA, pre versus test: F1; 19 = 0.65, *P*=0.46; drug versus saline: F1; 19 = 0.08, *P*=0.92, interaction: F1; 19 = 0.42, *P*=0.82; *n* = 5). (b) No difference was detected in the preference times of the sham group (*t*-test, *P* > 0.05). (c) No place preference was detected after the application of gabapentin (50 mg/kg) to the mice of SMIR (two-way RM ANOVA, pre versus test: F1; 31 = 0.09, *P*=0.69; drug versus saline: F1; 31 = 7.09, *P*=0.26, interaction: F1; 31 = 7.76, *P*=0.24; *n* = 8). (d) No difference was detected in the preference times of the SMIR group (*t*-test, *P* > 0.05). ANOVA, analysis of variance; RM, repeated measures; PI, preference index.

Gabapentin is the first-line medicine for the treatment of neuropathic pain. We further evaluated the analgesic effects of gabapentin on both evoked pain and spontaneous pain induced by SMIR treatments. As shown in Figure 5(a), saline application had no effect on the PWTs; the PWTs of mice remained unchanged after application of gabapentin at 10 mg/kg (Figure 4(b)), while the PWTs increased at 20 mg/kg (Figure 4(c)) and 50 mg/kg (Figure 4(d)), tested at 0.5 hours after the injection. This suggested that evoked pain could be relieved by gabapentin at doses of 20 mg/kg and 50 mg/kg. Similarly, we evaluated the analgesic effects of gabapentin on spontaneous pain after SMIR. As shown in Figures 6(a) and 6(b), the mice exposed to sham treatments did not show place preference to the gabapentin-paired chamber. Unlike our previous observations, the application of gabapentin at 50 mg/kg did not induce a preference for its paired chamber (Figure 6(c)); consistently, no difference was detected in the preference time (Figure 6(d)), which indicated that the gabapentin treatment failed to induce place preference. These data suggested that the spontaneous pain was not relieved by 50 mg/kg gabapentin on day 4 after SMIR.

## 4. Discussion

As mice are used for various gene knockout/down models, which allow further study of the mechanism underlying of pain. Our results demonstrated that the SMIR model in mice was developed and characterized by a significant decrease in withdrawal threshold to von Frey filaments for 14 days after the procedure and a spontaneous pain measured by CPP. Clonidine and gabapentin could relieve mechanical hypersensitivity dose-dependently. Moreover, clonidine but not gabapentin could alleviate the spontaneous pain of SMIR in the mice model. To our knowledge, this is the first study expanding the SMIR model from rat to mice successfully and investigating the effects of clonidine and gabapentin on spontaneous pain and mechanical allodynia simultaneously in mice SMIR model.

A rat model of SMIR developed by Flatters [10] was usually used for investigating the underlying mechanism of postsurgical pain [11–13]. Our study expanded this model from rat to mouse. Similar to the rat model by Flatters, our mice model showed hypersensitivity to mechanical stimulation but not heat stimulation. However, the two main differences between the two models were the summit and duration of pain behavior. Mechanical hypersensitivity evoked by SMIR in the rat model was observed by postoperative day 3, most prominent between postoperative days 10 and 13, lasted until at least postoperative day 22, and had dissipated by postoperative day 32. SMIR-evoked mechanical hypersensitivity of our mice model began on day 1 after the procedure, prominent between days 1 and 10 after the procedure, persisted at least until day 14, and disappeared on day 18 after the procedure. Another main difference between the two models was spontaneous pain behavior measured by CPP in our mice model. Allodynia and hyperalgesia, but not spontaneous pain, are frequently used to evaluate pain stages in animal models [10]. However, it has been reported that spontaneous pain is a major problem for patients with persistent pain [6–9]. Castel et al. developed a porcine model of postoperative pain with spontaneous behavior score [26]. However, a porcine model for studying the underlying mechanism is not commonly accepted. The presence of spontaneous pain has previously been reported and evaluated using the CPP behavioral assay [24]. Critical to such a study paradigm is the selection of drugs that do not possess rewarding properties in naive animals and their administration at sites that are not a part of the reward circuit. In our study, we employed clonidine, which is not known to be rewarding in naive mice. Moreover, we verified in our experiments that these agents did not produce CPP in saline-treated, sham-operated mice. On the other hand, clonidine produced robust CPP, which suggested the presence of ongoing spontaneous pain in these mouse models of persistent pain.

Recent studies have shown that some drugs have different effects on spontaneous pain and hyperalgesia, which suggested that different mechanisms may be involved in the regulation of spontaneous pain or evoked pain [27]. Our data suggested that clonidine at both 0.1 mg/kg and 0.5 mg/kg (i.p.) can relieve persistent allodynia, which is consistent with the previous report [24]. Similar to clonidine, gabapentin at both 0.1 mg/kg and 0.5 mg/kg (i.p.) can relieve persistent allodynia. However, CPP was induced by clonidine but not gabapentin in the mice model of SMIR. Our team had also found that the mechanical allodynia could be alleviated by the application of clonidine. Clonidine-induced place preference was only observed at day 7 in the mice model of common peroneal nerve [19]. Clonidine is reported to inhibit spinal LTP while gabapentin targets at voltage-gated calcium channels [28, 29]. It is likely that evoked pain and spontaneous pain are regulated by different mechanisms or by different analgesic drugs [30].

The limitation of this study was that the underlying mechanism, including acute pain transiting to chronic pain and the different effects of clonidine and gabapentin on CPP or evoked pain, has not been investigated. Gabapentin did not induce place preference or alleviate spontaneous pain, but attenuate evoked pain with high doses. Whether gabapentin combined with other analgesic drugs could alleviate both evoked pain and spontaneous pain should be investigated further in basic research and clinical trials.

In conclusion, the SMIR model of mice was developed successfully with a postoperative persistent pain syndrome, including evoked pain and spontaneous pain. Clonidine and gabapentin can both relieve mechanical hypersensitivity dose-dependently, but have different effects on spontaneous pain in our SMIR model. The underlying mechanism should be further investigated.

## Data Availability

The data used to support the findings of this study are available from the corresponding author upon request.
